# Brain’s Best Kept Secret: Degeneracy

**DOI:** 10.1523/ENEURO.0430-23.2023

**Published:** 2023-11-14

**Authors:** Christophe Bernard

**Affiliations:** Aix Marseille Univ, Inserm, INS, Institut de Neurosciences des Systèmes, Marseille, France

## Introduction

Neuroscientists endeavor to unravel the mysteries of brain functions and dysfunctions. A common research strategy involves measuring specific parameters across various conditions. These measurements are then typically repeated, averaged, and used to infer general patterns or rules. The act of averaging data is an ancient practice; for instance, early astronomers in Babylonian, Chinese, and Indian cultures implicitly averaged observations of celestial phenomena to predict significant periods, such as those crucial for agriculture.

Averaging is a sound approach when the process being studied follows to a mathematical function, represented as y = f(x), where f is a very general function. This is true even if the exact function is not known at the outset of the experiments. Implicit in this method is the assumption that any variations in measurements arise from imperfections in the recording process since a consistent mathematical rule suggests that identical inputs should always yield the same output. In essence, every time we encounter x1, we expect to measure y1.

However, a pervasive assumption in neuroscience is that everything we measure conforms to the rule y = f(x). This assumption overlooks a fundamental concept inherent to life: degeneracy. Degeneracy refers to the occurrence of different processes or structures leading to the same result. Take the function y = f(x), where f is the square root. The equation √4 yields two answers: −2 and +2. This dual solution exemplifies degeneracy. Square the two values, and you get 4. Two different processes lead to the same result.

Imagine we have a machine designed to compute square roots, but it lacks precision. Whenever it calculates √4, it might produce different results such as 2.01, 1.99, −2.08, and so on. If we were to average these results, we would get a value close to 0. This average obscures the real phenomenon, where half the values cluster around −2.0 and the other half around 2.0.

I shall come back to the problem of averaging in neuroscience later. Before addressing the problem of degeneracy in neuroscience, a historical perspective will show where degeneracy comes from and why it is important.

## Understanding Degeneracy

The field in which degeneracy was first described would be atomic physics or quantum mechanics, primarily in the early decades of the 20th century, notably by Niels Bohr and by Erwin Schrödinger. The phenomenon was observed before a full theoretical framework (quantum mechanics) was in place to explain it, with the use of the term solidifying as the theory developed. In the hydrogen atom, the 2s and 2p electron orbitals are degenerate because they have the same energy. However, they represent different spatial distributions of the electron. In the presence of a magnetic or electric field, this degeneracy disappears.

The concept of degeneracy, while deeply theoretical at its core, has contributed to technological and medical advancements that have had a tangible impact on human life.

- Semiconductor technology and electronics: The understanding of energy band structures, including degenerate energy levels, in solids laid the foundation for semiconductor physics. Semiconductors are the backbone of modern electronics. Degenerate semiconductors are used to build solar cells.
- Magnetic resonance imaging (MRI): The phenomenon behind MRI is nuclear magnetic resonance. The degeneracy of nuclear spin states is lifted when subjected to an external magnetic field, allowing for resonance absorption.
- Laser technology: The principle behind lasers involves the stimulated emission of radiation. The energy level structure, including degenerate states, of materials determines their suitability for specific laser types.
- Quantum computing: The construction of quantum computers involves qubits in superpositions of degenerate quantum states.

While the concept of degeneracy has its roots in theory and had a profound impact on various fields, one might wonder: does degeneracy also apply to biology?

## Degeneracy in Biology

The classical example is the genetic code. Multiple codons can encode for a single amino acid. This is genetic degeneracy. In neuroscience, it means that multiple pathways, often structurally distinct, can produce the same functional output.

**Figure 1.**
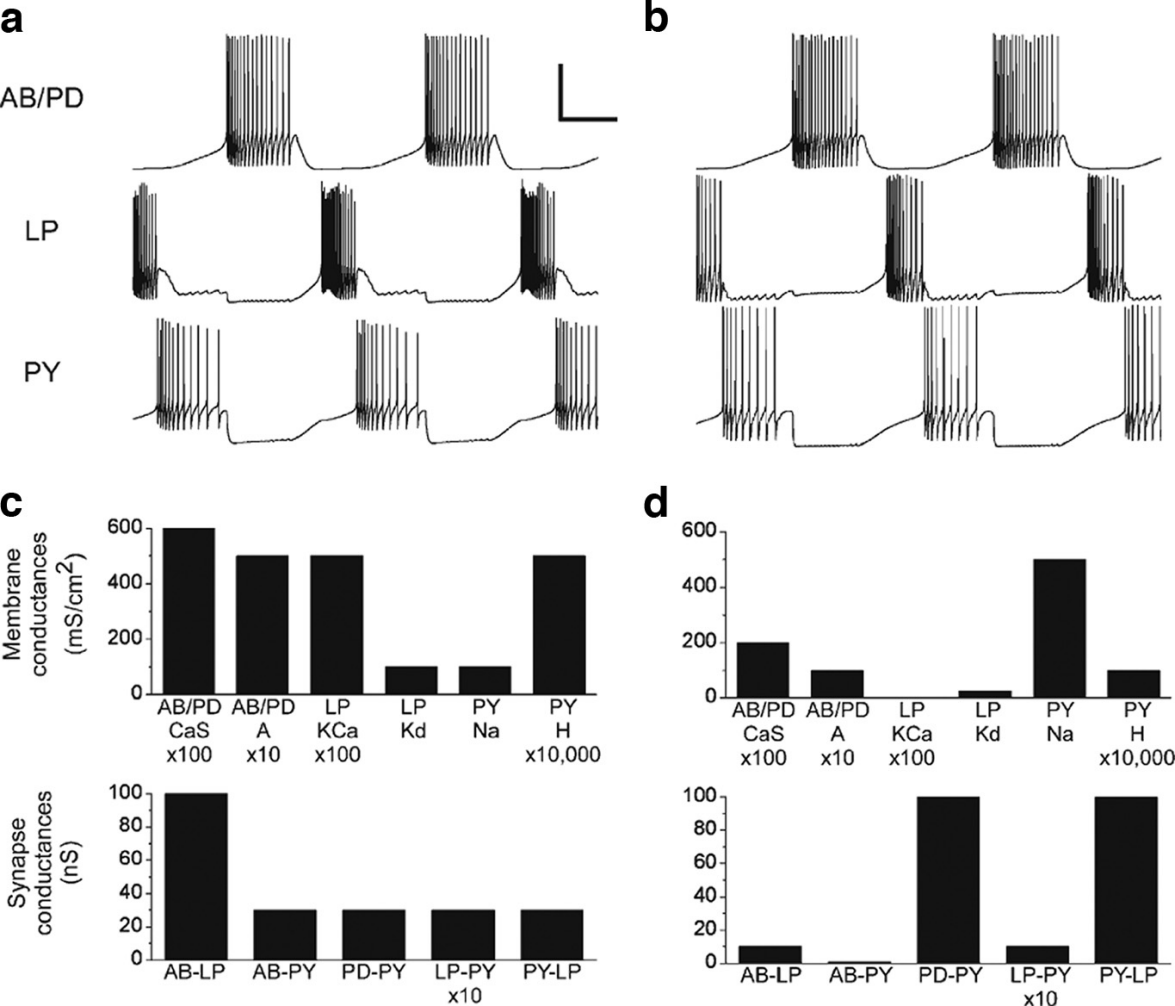
Similar model-network activity from different network properties. The pyloric rhythm is produced by a pacemaker network consisting of the anterior burster (AB) neuron, which is electrically coupled to two pyloric dilator (PD) neurons, and of two types of follower neurons: a single lateral pyloric (LP) neuron and five to eight pyloric (PY) neurons. The follower neurons receive inhibitory glutamatergic synapses with fast dynamics from the AB neuron, and inhibitory cholinergic synapses with slow dynamics from the PD neurons. The LP neuron has an inhibitory glutamatergic synapse that feeds back to the PD neurons, and the LP neuron has reciprocal inhibitory glutamatergic connections with at least some of the PY neurons. ***a***, ***b***, Voltage traces from two model pyloric networks. Scale bars: 0.5 s and 50 mV. ***c***, Selected membrane conductances (top) and synaptic conductances of the model network shown in ***a***. ***d***, Selected membrane conductances (top) and synaptic conductances of the model network shown in b. The two networks generated very similar activity despite very different cellular and synaptic properties. Reprinted with permission from Prinz et al. (2004).

Gerald Edelman demonstrated degeneracy within the immune system and proposed the generalization of the concept to biology in general and neuroscience in particular (Tononi et al., 1999; Edelman and Gally, 2001). Eve Marder’s combined computational and experimental work provides an excellent illustration of degeneracy in neuroscience (Prinz et al., 2004). In Figure 1, two different neuronal networks are constructed *in silico*. Both generate exactly the same output in terms of firing pattern. Although the networks have completely distinct ion channel and synapse properties, they exhibit the same activity. This exemplifies degeneracy. Experimental recordings in the pyloric system confirmed that animals exhibit the same rhythmic activity although they have varying structural differences.

These examples underscore that degeneracy is not just a theoretical construct but a fundamental aspect of life, driving diversity in biological systems while preserving functionality. Why is degeneracy beneficial from a biological standpoint?

## Degeneracy: Essential to Life

- Evolutionary advantage: degeneracy can confer evolutionary benefits. When different systems have the capacity to fulfill the same function, it offers a safety net against unpredictable environmental changes, thereby promoting survival. As an example, Eve Marder’s research reveals that the rhythmic activity she investigates remains stable under rising temperatures for certain animals, but not for others (Haddad and Marder, 2018). This variability is rooted in the temperature sensitivity of chemical reactions and biophysical properties of molecules, with each animal possessing a distinct internal architecture that determines its specific temperature responsiveness.
- Flexibility and resilience: while the adaptability of the brain is commonly attributed to its plastic nature, degeneracy could be a key contributor to this property. When parts of a neural network face disruption, for example, due to aging, a reconfiguration of the internal structure might take place to counteract this disturbance. For illustrative purposes, consider Figure 1. If we start with the network in the left configuration and then encounter a block in the transcription of the KCa channel in LP neurons, the network could transition to the right configuration, preserving its rhythmic activity without alteration.
- Complex dynamics and redundancy: the complexity of the brain means that a mere inventory of its components is not sufficient for a comprehensive understanding. Its functionalities are emergent, shaped by interactions, feedback mechanisms, and nonlinear dynamics. In this intricate dance of processes, degeneracy plays a crucial role by ensuring that these interactions remain undisturbed even when specific components are compromised.

### Degeneracy and the brain

The inherent degeneracy of biological systems may be a key reason why profound insights into the brain remain tantalizingly out of reach.

Consider the illustration in Figure 1. It challenges the oversimplified notion that the relationship in living organisms is just y = f(x). Instead, it suggests a more intricate y = f_i_(x), where “i” denotes a specific organism. Here, f_i_ embodies the unique set of parameters that define an individual “i,” ensuring that f_i_ is distinct from f_j_ across any two organisms, “i” and “j.” In the context of Figure 1, f_left_ drastically differs from f_right_, which has implications for data interpretation.

In trying to understand brain function, let us consider an illustrative scenario using the network depicted in Figure 1. Imagine we are focused on the I_KCa_ current and hypothesize its role in modulating oscillations. We proceed to test this hypothesis in various animals using a specific channel blocker. The results vary, as is often the case in biological experiments. On average, the blocker might reduce oscillation amplitude by X ± Y%. However, the nuanced role of I_KCa_ cannot be isolated from its broader context in the network. The action and influence of each component are interrelated and contingent on the system as a whole. Such variability in the effects of the I_KCa_ blocker in different brains is a testament to the principle of degeneracy. In some configurations, like the one on the right of Figure 1, the blocker may even prove ineffective.

Thus, degeneracy provides a conceptual lens to elucidate the variability in treatment responses across individuals and the differential responses seen in pathologic scenarios.

The unique internal structure (represented by f_i_) observed across animals may extend to networks in which the organization appears repetitive. In the cortex, neurons appear regularly organized in distinct layers along the *z*-axis and seemingly repeating cellular configurations on the *x* and *y* planes. It is plausible that two neurons in the same plane, say two neighboring layer 5 pyramidal cells, though appearing identical, possess fundamentally different internal structures, such as different sets of ion channels, yet still perform identical functions, echoing the principle of degeneracy.

The principle of degeneracy holds true across different scales. For example, systematically knocking out genes in unicellular organisms can produce subtle functional effects (Winzeler et al., 1999). This means that the organism can still find a stable solution despite the lack of a specific gene. The right panel of Figure 1 could correspond to an animal in which the gene coding for the protein making I_KCa_ has been knocked out. Yet, the activity is at it should be, because a f_i_ exists that account for the absence of I_KCa_. At the other end of the spectrum, if we consider where/how information is processed in the brain, neurons in nearly all regions respond nonspecifically when a mouse initiate an action (Steinmetz et al., 2019). However, this holistic view should not be misconstrued to imply that every component is equally important. Like genes, certain elements might be indispensable. If they are absent, there might not be an equivalent function f_i_ that compensates for their absence. This insight is underscored by research on systematic gene knock-downs (Winzeler et al., 1999).

## Is Degeneracy Hindering Major Breakthroughs?

The essence of the scientific method hinges on repeated observations aimed at distilling a universal principle. Such an approach assumes the subject under study exists within a stable context. Take astronomy, for instance: its immutable laws, such as gravitation, serve as this constant backdrop. Repeated observations, in this context, help pinpoint variabilities attributed to measurement inaccuracies.

Biological systems introduce a different kind of variability, one that is not solely because of measurement errors and the temporal variability in time of the properties of organisms, but is deeply rooted in the principle of degeneracy.

In neuroscience, researchers implicitly assume that the parameter under investigation is so fundamental to a given function that any inherent variability, potentially resulting from degeneracy, will not significantly skew data interpretation or hinder the extraction of general principles. But not all neuronal parameters bear the criticality of, say, ribosomal proteins, which are truly indispensable. It is quite plausible that a significant proportion of brain parameters resemble the I_KCa_ from the illustrative example. These parameters may exhibit vast variations across individual neurons and brains. Such variability is possible because multiple configurations of neurons and networks can support these differences while still achieving analogous functionalities.

### Is averaging misleading in neuroscience?

Averaging data, while a common practice, can often lead to misinterpretations in neuroscience. The crux of the problem is that the value of a given parameter is inherently contextual (as denoted by f_i_). Without understanding this context, interpreting the values becomes problematic.

Consider a scenario in which a brain is observed in two different conditions: a standard or “control” setting and a pathologic one. A typical observation is that significant differences emerge between these conditions. Suppose we use three variables to characterize each brain, allowing us to represent each brain as a point in a three-dimensional space defined by these variables. Assume that all configurations corresponding to normal vision lie on the surface of the sphere. However, researchers typically lack this overarching context (we do not know that normal vision spans the surface of the sphere, which contains all f_i_ contexts).

In examining “normal” individuals, data points cluster near the north pole of the sphere. When observing individuals with epilepsy (who generally do not suffer visual impairments because of the condition), the data points gravitate toward the south pole. If these datasets were averaged and compared, the statistical differences would be striking. Yet, this interpretation is misleading, as both datasets are part of the same broader distribution associated with normal vision. The real challenge is our ignorance of the true shape of this overarching distribution.

A more nuanced interpretation might suggest that the different data distributions showcase the principle of degeneracy. It is possible that individuals without any pathology predominantly reside near the north pole. However, epilepsy might induce multiple internal parameter shifts. Because of degeneracy, the existence of multiple pathways to achieve the same outcome, a stable context for normal vision could re-emerge near the south pole. Thus, observed network alterations in pathologic conditions could be mere reflections of degeneracy and may not directly correlate with the observed symptoms.

### Accounting for degeneracy in neuroscience research

Degeneracy, although often overlooked, has profound implications in neuroscience. Specifically:

1. It poses challenges by mandating the consideration of individual variability.
2. It unveils a vast spectrum of possibilities, thereby broadening the scope of research.
3. It prompts introspection about our current research methodologies in neuroscience.

Given that the concept of degeneracy in biology was introduced nearly a quarter-century ago, its incorporation into our research practices is long overdue. It is high time that we stop treating it as a mere anomaly. So, how can we address this?

1. Acknowledge and educate: the foundation is awareness. Neuroscientists must grasp the nuances of degeneracy and how it impacts their research. This necessitates comprehensive educational initiatives at every academic stage. Personally, I prioritize degeneracy in my M.S. and Ph.D. course curricula.
2. Individualized data collection: move away from a one-size-fits-all approach. Instead of making generalized assumptions based on averages, focus on individual variations. This may require collecting more detailed data on individual subjects, which may be facilitated by recent advances in data collection technologies. A prime example is the growing emphasis on personalized medicine, such as the Virtual Epileptic Patient initiative aiming to virtualize individual brains for surgical decision-making (Jirsa et al., 2017).
3. Statistical methods: can conventional statistical approaches truly accommodate degeneracy? Perhaps it is time to innovate?
4. Modelling and simulations: use computer models to simulate brain activity. These models can help in understanding how different parameter distributions can lead to similar outcomes and how changing one parameter can affect the system as a whole.
5. Longitudinal studies: observing changes over time can offer insights into how degeneracy plays a role in aging or disease processes. For instance, how does the brain compensate for the loss of certain functions over time?
6. Focus on pathological conditions: by studying conditions in which the normal functioning of the brain is disrupted (such as epilepsies, Alzheimer’s disease, or stroke), we can glean insights into how the brain adapts and which alternative pathways it uses to still perform essential functions despite reorganization.
7. Interdisciplinary collaboration: fields like physics and mathematics, which already factor in degeneracy, can offer fresh perspectives. We may even need to craft new mathematical constructs tailored to biology to adequately encapsulate degeneracy.
8. Embrace heterogeneity: variability is not noise, it is data. By valuing this heterogeneity, we can garner richer datasets and deeper interpretations.

By adopting these strategies, neuroscience can better account for the inherent degeneracy of biological systems, leading to more accurate predictions, better interventions, and a deeper understanding of the brain’s complexities. According to J. B. S. Haldane, there are four stages of acceptance for scientific ideas:

1. this is worthless nonsense;
2. this is an interesting, but perverse, point of view;
3. this is true, but quite unimportant;
4. I always said so.

In neuroscience, we appear to be stuck at the second stage. We may consider degeneracy as a perverse point of view because it makes our life more complicated and because it questions the way we do science. The earlier we reach stage four, the better it will be for our understanding of the brain.

## Conclusion

At its core, degeneracy is not an oddity, it is central to the architecture and operation of the brain. Truly understanding it may be our golden ticket to deciphering the brain’s enigmas. If we aim to fully demystify the workings of the brain, recognizing and capitalizing on degeneracy is indispensable. Consider the profound insights and transformative changes brought about by understanding degeneracy in quantum physics. Now, imagine the monumental strides we could make in neuroscience by fully integrating the concept of degeneracy. Perhaps, in the grand cerebral puzzle, degeneracy is the elusive piece we have been hunting. Degeneracy, the brain’s best-kept secret, may in fact be its most powerful.

## Synthesis

Reviewing Editor: Christophe Bernard, INSERM & Institut de Neurosciences des Systèmes Decisions are customarily a result of the Reviewing Editor and the peer reviewers coming together and discussing their recommendations until a consensus is reached. When revisions are invited, a fact-based synthesis statement explaining their decision and outlining what is needed to prepare a revision will be listed below. The following reviewer(s) agreed to reveal their identity: NONE.

N/A
